# Evaluation of sociodemographic, clinical and behavioral characteristics of people living with the human immunodeficiency virus and its association with quality of life

**DOI:** 10.1590/1980-549720250034

**Published:** 2025-06-27

**Authors:** Ehideé Gómez La-Rotta, Leidy Janeth Erazo Chavez, Harold Gomez-Larrota, Pedro Henrique de Faria, Armindo Augusto da Nobrega Albuquerque, Felipe Thiele Cecílio, Max da Silva Maciel, José Antonio Enciso Domínguez, Maria Rita Donalisio

**Affiliations:** I Universidade da Integração Latino-Americana, Public Health Program – Foz do Iguaçu (PR), Brazil.; II Universidade Federal do Maranhão, Graduate Program in Public Health – São Luís (MA), Brazil.; III Universidade Ceuma, Medicine and Psychology Program – São Luís (MA), Brazil.; IV NTS Electronic Control – Bogotá, Cundinamarca, Colombia.; V Universidade Estadual de Campinas, School of Medical Sciences, Graduate Program in Public Health – Campinas (SP), Brazil.; VI Universidade de São Paulo, Hospital de Clínicas – São Paulo (SP), Brazil.

**Keywords:** Quality of life, HIV long-term survivors, Race factors, Sex, Inequalities

## Abstract

**Objective:**

To investigate inequalities of race/skin color and sex in relation to quality of life of people living with the human immunodeficiency virus (HIV) in a Brazilian university hospital between 2017 and 2018.

**Methods:**

This is a cross-sectional study conducted between 2017 and 2018 with 350 people living with HIV, applying the HIV-specific Quality of Life (QoL) scale. The groups were compared using the χ^2^ test and Student's *t*-test or Kruskal-Wallis test. To evaluate the factors associated with the nine domains of QoL, Tweedie Regression, an application of the Generalized Linear Model, was performed.

**Results:**

Of the 350 participants, 55.7% self-reported to be white and 44.3%, Black/mixed-race; with a mean age (standard deviation – SD) of 45.2 (±12.6). We verified that 46.3% reported having suffered some type of prejudice, 34.8% due to the disease, 12% due to skin color, and 11.4% due to sexual orientation. The overall mean quality of life was 78.85 (±11.61). The domain with the lowest mean was Confidentiality Concerns (M: 43.45±29.46). Among the factors associated with several domains were sex (woman), level of education, per capita income, having suffered some prejudice, or hospitalizations in the last year.

**Conclusion:**

Differences in level of education, per capita income, and work status by self-reported race/skin color of the participants were evidenced, showing inequalities in the study population. Sex (woman) is the factor associated with most of the QoL domains, among them Life Satisfaction, Confidentiality Concerns, Health Concerns, Financial Concerns, Medication Concerns, Acceptance of HIV, and Sexual Function.

## INTRODUCTION

In 2023, 39 million people worldwide lived with the human immunodeficiency virus (HIV)/acquired immunodeficiency syndrome (AIDS), a number that continues growing due to the increase in survival related to access to antiretroviral therapy (ART)^1^.

In Latin America, the number of new HIV cases in 2022 was 110 thousand, although the number of deaths decreased^1,2^. In Brazil, 43,403 cases of HIV infection were reported, and 6,759 (44.6%) were diagnosed in the state of São Paulo. However, the mortality rate in Brazil decreased by 26.5% between 2012 and 2022^3^.

Although there is a well-organized national program to combat AIDS in Brazil, it should be noted that economic and political aspects have a significant effect on each population^4^. Most cases are located in poor countries, suggesting the need to analyze social determinants and inequalities in these populations^5^.

In this sense, there was an increase in the incidence of HIV cases in Brazil among vulnerable populations and those of lower socioeconomic status, including mixed-race/Black individuals, accentuating structural racism and the vulnerability conditions of this population^3,6^. The proportion of HIV infections among mixed-race/Black people was 60.1% (48.4% of mixed-race individuals and 11.7% of Black individuals); and among white people, 33.1% in 2022, and deaths occurred in 61.7% (47.0% of mixed-race people and 14.7% of Black people) and 35.6% among white people^3^.

With the increase in survival rates of people with HIV/AIDS, which characterizes it as a chronic disease, the health care provided to this group gains greater importance due to the need for unique care to maintain their quality of life (QoL)^4^.

Our objective in this study was to investigate inequalities in race/skin color and sex in relation to the QoL of people living with HIV in a Brazilian university hospital.

## METHODS

A cross-sectional study was conducted on people living with HIV (PLHIV) whose self-reported skin color was mixed-race/Black and white, followed up at the outpatient clinic of infectious diseases of Hospital das Clínicas of Universidade Estadual de Campinas (HC-Unicamp)^1^ between October 2017 and April 2018. The research was approved by the Research Ethics Committee (REC), under opinion No. 2.148.789, of August 2017.

Considering an HIV detection rate of 17.8 cases per 100 thousand inhabitants in Brazil in 2019, the minimum number of individuals was calculated. Considering the population of 4,900 PLHIV in follow-up in 2015, with a 5% maximum sampling error and 95% confidence interval, a minimum sample of 357 individuals was estimated.

Patients’ recruitment was performed by weekly systematic recruitment and direct contact during attendance in appointments, over eight months of data collection, with selection according to the following criteria: HIV positive patients, over 18 years old, followed up in the outpatient clinic of infectious diseases, and who did not suffer from any cognitive deficit that prevented them from following the guidelines.

The interviews were conducted face-to-face on the day of the patient's appointment, in a reserved location. Subsequently, their medical records were analyzed to obtain information on clinical variables (classification of clinical status, CD4 cell count, viral load, hospitalizations in the last year, causes of hospitalizations, adherence to treatment^2^, medications and doses) on the day of the interview or months later, respecting what was approved and provided for in the REC.

Information was collected by filling out a physical questionnaire, with five sections in which sociodemographic characteristics were investigated as follows: self-reported skin color, age, sex, marital status, level of education, per capita income (minimum wage — BRL 937; USD 293, currency for 2018), religion, work status, sexual orientation, whether the patient suffers prejudice (due to skin color, sex, being a carrier of HIV/AIDS, sexual orientation), risk behavior for HIV infection, clinical evolution, and disease severity.

To evaluate QoL, the Quality of Life Questionnaire (HAT-QoL)^7,8^ was used, validated for Brazilian Portuguese^9^ and composed of 34 items that evaluate nine domains of QoL: General Function (GF), Life Satisfaction (LS), Health Concerns (HC), Financial Concerns (FC), Medication Concerns (MC), Acceptance of HIV (AHIV), Confidentiality Concerns (CC), Confidence in the professional (CP), and Sexual Function (SF).

To answer each question, individuals were led to think about their quality of life in the last four weeks. The answers had a five-point Likert scale format. In each domain, 0 is the lowest score and 100 is the best possible score. The higher the score, the lower the impact of HIV infection on the QoL of individuals, and vice versa^9^.

The database was fed into an Excel spreadsheet by Google resources. Subsequently, it was transferred and analyzed using the Statistical Package for the Social Sciences (SPSS), version 21.0 (SPSS, Chicago, IL).

The proportions of categorical variables and the measures of central tendency and dispersion for continuous variables were calculated. Categorical variables were compared with χ^2^ and Fisher's exact tests, the means with Student's *t*-test for independent samples, and the median with Kruskal-Wallis. The value of p<0.05 was considered as a marker of statistical difference in all analyses.

The outcome variables were the QoL domains and the exposure variables were self-reported skin color and sex, in addition to other sociodemographic, clinical, access, and prejudice variables. To determine the correlation between sociodemographic and clinical variables and QoL domains evaluated with HAT-QoL, the Pearson's method was used^10^. However, the results found in this research show little correlation between sociodemographic and clinical variables and QoL domains (Appendix 1).

To evaluate the association of the QoL domains with the collected covariables (independent variables), the Tweedie regression, an application of the Generalized Linear Model, was performed. The univariate analysis was carried out first, which allowed us to select the variables that presented a value of p<0.20 for the multiple model construction. The variables that remained in the final multiple model were those that presented statistical significance (p<0.05) in the backwards procedure.

In Tables 1 and 2, the mixed-race/Black skin color populations were grouped, as recommended by the Brazilian Institute of Geography and Statistics (IBGE)^11^; and white and Asian skin color populations were grouped in the other category.

**Table 1:** Sociodemographic and behavioral characteristics of patients seen at the HIV/AIDS outpatient clinic in relation to self-reported race/skin color, Campinas (SP), 2017–2018.

Variables		White skin color (155)	Mixed-race/Black skin color (195)	Total (350)	p-value
		f (%)	f (%)	f (%)	
Age					
	Median (25.75)	48 (39.55)	43 (36.50)	45 (37.53)	0.013^§^
	Mean±SD	47.12 (12.9)	43.7 (12.2)	45.2 (12.6)	
	Minimum–maximum	20–6	18–76	18–76	
Sex					
	Man	97 (62.6)	108 (55.4)	205 (58.6)	0.262^‡^
	Woman	55 (35.5)	85 (43.6)	140 (40.0)	
	Transgender; other	3 (1.9)	2 (1.0)	5 (1.4)	
Marital status					
	Single/separated/widowed	100 (64.5)	116 (59.5)	216 (61.7)	0.376^†^
	Married/Common-law marriage	55 (35.5)	79 (40.5)	134 (38.3)	
Level of education					
	Median (25.75)	10 (8.15)	9 (4.10)	9 (4.12)	0.013^§^
	Mean±SD	10.2 (4.56)	8.56 (3.84)	9.29 (4.24)	
	Minimum–maximum	4–19	0–19	0–19	
Per capita income*					
	Median (25.75)	937 (465.2000)	505 (312.1000)	666 (357.1400)	0.001^//^
	Mean±SD	1,506 (1602)	927 (1403)	1,181 (1519)	
	Minimum–maximum	0–10,000	0–11,666	0–11,666	
Sexual orientation					
	Heterosexual	98 (63.2)	159 (81.5)	257 (73.4)	0.001^†^
	Homosexual	47 (30.3)	29 (8.3)	76 (21.7)	
	Other (Bisexual)	10 (6.5)	7 (2.0)	17 (4.9)	
Number of partners*					
	Median (25.75)	1 (1.1)	1 (1.1)	1 (1.1)	0.754^//^
	Mean±SD	1.92 (4.73)	1.57 (2.84)	1.73 (3.80)	
	Minimum–maximum	0–50	0–30	0–50	
Use of condoms*					
	Yes	103 (65.5)	149 (76.4)	252 (72.0)	0.002^†^
	No	23 (14.8)	18 (9.2)	41 (11.7)	
	Not applicable	29 (18.7)	28 (14.4)	57 (16.3)	
Recreational drugs					
	Yes	79 (51.0)	85 (43.6)	164 (46.9)	0.196^†^
	No	76 (49.0)	110 (56.4)	186 (53.1)	
Type of drugs^¶^					
	Alcohol (yes)	52 (34.4)	57 (30.5)	109 (32.2)	0.512^†^
	Tobacco (yes)	34 (23.8)	48 (25.8)	82 (24.9)	0.673^†^
	Marijuana (yes)	10 (6.6)	2 (1.1)	12 (3.5)	0.006^‡^
	Crack cocaine (yes)	1 (0.7)	5 (2.6)	6 (1.8)	0.334^‡^
	Cocaine (yes)	1 (0.7)	2 (1.0)	3 (0.9)	0.539^‡^
	Others (yes)	2 (1.3)	0 (0.0)	2 (0.6)	0.069^‡^

HIV: Human immunodeficiency virus; SD: standard deviation.

* Non-normal distribution — Shapiro-Wilk (0.001);

p of categorical variables: χ^2†^ and Fisher's^‡^ exact tests. p of discrete variables: Student's *t* ^§^ and Kruskal-Wallis^//^; ^¶^data of those who are currently using it.

**Table 2:** Access and clinical aspects of patients seen at the HIV/AIDS outpatient clinic in relation to self-reported race/skin color, Campinas (SP), 2017–2018.

Variables		White skin color (155)	Mixed-race/Black skin color (195)	Total (350)	p-value
		f (%)	f (%)	f (%)	
Form of access					
	Emergency care services	45 (31.5)	70 (37.2)	115 (34.7)	0.007^‡^
	SUS/UBS	35 (24.5)	61 (32.4)	84 (29.0)	
	Physician/Health insurance plan	31 (21.7)	14 (7.4)	45 (13.6)	
	CAISM	14 (9.8)	16 (8.5)	30 (9.1)	
	Spontaneous search	6 (4.2)	11 (5.9)	17 (5.1)	
	Blood center	4 (2.8)	10 (5.3)	14 (4.2)	
	Others	8 (5.6)	6 (3.2)	14 (4.2)	
Diagnosis time*					
	Median (25.75)	13 (6.4–20.3)	12.3 (5.3; 19)	12.3 (5.9; 19.3)	0.209^//^
	Mean±SD	13.4 (8.02)	12.3 (12.2)	12.78 (7.9)	
	Minimum–maximum	0.59–34.2	0.43–33.3	0.43–34.2	
Treatment time*					
	Median (25.75)	11.1 (5.1–19.4)	10.7 (4.2–18)	10.9 (4.8–18.1)	0.354^//^
	Mean±SD	11.9 (7.72)	11.2 (7.56)	11.5 (7.63)	
	Minimum–maximum	0.20–27.5	0.11–28.0	0.11–28.0	
Difference between Dx and treatment onset*					
	Median (25.75)	0.08 (0.01; 1)	0.08 (0.01; 0.5)	0.08 (0.0–0.7)	0.482^//^
	Mean±SD	1.59 (3.63)	1.14 (2.76)	1.34 (3.18)	
	Minimum–maximum	3.33–19	0.01–16	3.33–19	
Clinical status^¶^					
	CDC-A	68 (43.9)	74 (37.9)	143 (40.6)	0.230^†^
	CDC-B	31 (20.0)	32 (16.4)	63 (18.0)	
	CDC-C	56 (36.1)	89 (45.6)	145 (41.4)	
CD4 cel./mm^3^					
	<200	9 (5.8)	15 (7.8)	24 (6.9)	0.771^†^
	200–499	51 (33.1)	63 (32.8)	114 (32.6)	
	>500	94 (61.0)	114 (59.4)	208 (60.1)	
Viral load^#^					
	<50	138 (90.2)	167 (86.1)	305 (87.9)	0.442^†^
	51–10,000	10 (6.5)	20 (10.3)	30 (8.6)	
	>10,001	5 (3.3)	7 (3.6)	12 (3.5)	
Treatment					
	Yes	153 (98.7)	193 (99.0)	346 (98.9)	0.817^†^
	No	2 (1.3)	2 (1.0)	4 (1.1)	
Number of medicines					
	Median (25.75)	3 (1.0; 5.0)	3 (1.0; 3.0)	3 (1.0; 4.0)	0.229^§^
	Mean±SD	3.03 (2.23)	2.78 (1.82)	2.89 (2.01)	
	Minimum	0	0	0	
	Maximum	10	10	10	
Poor adhesion					
Yes		40 (25.8)	56 (28.7)	96 (27.4)	0.544^†^
No		115 (74.2)	139 (71.3)	254 (72.6)	
Hospitalizations in the last year					
	Yes	22 (14.2)	39 (20.0)	61 (17.4)	0.155^†^
	No	133 (85.8)	158 (80.0)	289 (82.6)	
Cause of hospitalization					
	HIV	11 (50.0)	24 (61.5)	35 (57.4)	0.382^†^
	Other causes	11 (50.0)	15 (38.5)	26 (42.6)	

HIV: Human immunodeficiency virus; SUS: Brazilian Unified Health System; UBS: Health Center; CAISM: Comprehensive Health Care Center; SD: standard deviation; Dx: diagnosis; CDC: Centers for Disease Control and Prevention; CDC-A: less-advanced disease; CDC-B: moderately-advanced disease; CDC-C: advanced disease.

* Non-normal distribution — Shapiro-Wilk (0.001), measured in years;

p of categorical variables: χ^2†^ and Fisher's^‡^ exact tests; p of discrete variables: Student's *t* ^§^ and Kruskal-Wallis^//^; ^¶^according to the criteria of the Centers for Disease Control and Prevention (CDC); ^#^copies/mL^3^.

## RESULTS

### Socioeconomic and demographic characteristics

We interviewed 350 patients, with a mean age (standard deviation — SD) of 45.2 (±12.6); 58.6% were men, 33.8% were married/in a common-law marriage, and 42.2% reported having an elementary school degree, with an average of 9.3 (±4.2) years of formal education. There were 195 (55.7%) white people and 155 (44.3%) mixed-race/Black people with differences in level of education, per capita income, and work status (p<0.001). Patients who reported being mixed-race/Black have low levels of education, low income, and different work statuses (Table 1).

We found differences among sexes: men had higher means of level of education and better income (Table 3).

**Table 3:** Sociodemographic and behavioral characteristics of patients seen at the HIV/AIDS outpatient clinic regarding sex, Campinas (SP), 2017–2018.

Variables		Women (140)	Men (205)	Total (345)	p-value
		f (%)	f (%)	f (%)	
Age					
	Median (25.75)	46 (37.54)	43 (36.50)	45 (37.53)	0.720^§^
	Mean±SD	45.79 (12.4)	43.7 (12.2)	45.2 (12.6)	
	Minimum–maximum	18–76	18–76	18–76	
Marital status					
	Single/separated	80 (55.2)	136 (66.3)	211 (61.7)	0.131^†^
	Married/Common-law marriage	65 (44.8)	69 (33.7)	134 (38.3)	
Level of education					
	Median (25.75)	9 (4; 10.5)	9 (4; 10)	9 (4; 12)	0.052^§^
	Mean±SD	8.76 (4.04)	9.96 (4.37)	9.29 (4.24)	
	Minimum–maximum	4–19	0–19	0–19	
Per capita income					
	Median (25.75)	500 (300; 900)	900 (450; 1667)	666 (357; 1400)	0.009^//^
	Mean±DP	891 (1515)	1,387 (1491)	1,181 (1519)	
	Minimum–maximum	0–11667	0–10000	0–11667	
Sexual orientation					
	Heterosexual	139 (95.8)	118 (57.6)	257 (73.4)	0.001^†^
	Homosexual	3 (2.1)	73 (35.6)	76 (21.7)	
	Other (Bisexual)	3 (2.1)	14 (6.8)	17 (4.9)	
Number of partners*					
	Median (25.75)	1 (0.1)	1 (1.2)	1 (1.1)	0.001^//^
	Mean±SD	0.74 (0.75)	2.42 (4.81)	1.73 (3.80)	
	Minimum–maximum	0–7	0–50	0–50	
Use of condoms*					
	Yes	87 (60.0)	165 (80.5)	252 (72.0)	0.001^†^
	No	14 (9.7)	27 (13.2)	41 (11.7)	
	Not applicable	44 (30.3)	13 (6.3)	57 (16.3)	
Recreational drugs					
	Yes	60 (41.4)	104 (50.7)	164 (46.8)	0.084^†^
	No	85 (58.6)	101 (49.3)	186 (53.1)	
Type of drugs^¶^					
	Alcohol (yes)	35 (24.5)	74 (37.9)	109 (32.2)	0.009^†^
	Tobacco (yes)	30 (21.6)	52 (27.4)	82 (24.9)	0.231^†^
	Marijuana (yes)	3 (2.1)	9 (4.6)	12 (3.5)	0.212^‡^
	Crack cocaine (yes)	2 (1.4)	4 (2.0)	6 (1.8)	0.662^‡^
	Cocaine (yes)	1 (0.7)	2 (1.0)	3 (0.9)	0.757^‡^
	Others (yes)	0 (0.0)	2 (1.0)	2 (0.6)	0.228^‡^

HIV: Human immunodeficiency virus; SD: standard deviation.

* Non-normal distribution — Shapiro-Wilk (0.001);

p of categorical variables: χ^2†^ and Fisher's^‡^ exact tests. p of discrete variables: Student's *t* ^§^ and Kruskal-Wallis^//^; ^¶^data of those who are currently using it.

### Behavioral and epidemiological characteristics

As described in Table 1, 73.4% reported being heterosexual and 59.1% had a steady partner, with a mean of 1.7 (±3.8) partner per year. We verified differences in the number of partners in the last year and in the use of condoms as a means of preventing infection between sexes (p<0.001). Men had a mean of 2.4 (±4.8), while women had a mean (SD) of 0.7 (±0.73) partner in the last year; and men used condoms more frequently (80.5%) than women (60.0%).

Regarding the use of recreational drugs, we observed that 164 (46.7%) use them, the following being more prevalent: alcohol, 109 (32.2%); tobacco, 82 (24.9%); and marijuana, 12 (3.5%). All of them are consumed more often by men (p<0.001).

### Access to the service and clinical characteristics

In Table 2, we present data on access and clinical aspects. By analyzing the form of access to the healthcare service, we found that the majority accessed it through the public health flows of the Brazilian Unified Health System (SUS) (77.6%). Conversely, white individuals significantly reported that the form of access was by referrals of private network or health insurance physicians, recommendation of patients undergoing treatment in the service, and spontaneous search (31.5%); self-reported mixed-race/Black people followed the SUS flow, having a higher proportion of referrals from public institutions (83.5%).

Among the study participants, the most prevalent clinical stages were asymptomatic (CDC-A), with 40.6%; and AIDS (CDC-C), with 41.4%. The means (SD) of CD4 lymphocyte count and viral load at the last appointment were 675.5 cel./mm^3^ (492), and viral load of 45.2 copies/mL of blood (12.6), respectively. Undetectable viral load was deemed <50 copies of RNA/mL.

Of the participants, 98.9% were under treatment, taking 2.9 (2.0) antiretroviral drugs per day on average (SD). Among the most prevalent drugs and therapy regimens we found tenofovir/lamivudine/efavirenz (TDF+3TC+EFZ), with 36.9%; tenofovir/lamivudine/atazanavir/ritonavir (TDF+3TC+ATV/R), with 19.1%; darunavir/ritonavir (DRV/R), with 21.4%, but always associated with another antiretroviral drug, especially TDF+3TC (4.28%), with no difference by self-reported race/skin color.

The percentage of hospitalizations for all causes not associated with HIV in the last year was 17.4%. Nevertheless, the prevalence of HIV-related hospitalizations was 57.4%.

When assessing factors associated with HIV-related hospitalizations, we found that patients who had poor adherence to treatment had a higher prevalence (73.1%) of HIV-related hospitalizations.

### Information on suffered prejudices

Among the participants, 182 (52.0%) reported having suffered some kind of prejudice in their lives, especially due to the HIV infection — 121 (34.6%). However, 175 (50%) did not report having suffered prejudice because "nobody knows about the disease," when it comes to diagnosis. There were significant differences in prejudice by self-reported race/skin color and sex.

### Quality of life and associated factors

The overall mean±SD of HAT-QoL in this sample was 78.85 (±11.61). The domains with the highest means were *Confidence in the* professional (M: 94.45±12.62) and *Health concerns* (M: 92.10±12.47). The domains *Confidentiality concerns* (M: 43.45±29.46) and *Financial concerns* (M: 64.62±30.52) had lower means (Table 4). To verify the internal validity of the HAT-QoL scale, the internal consistency of each domain was calculated, and we found good and excellent agreements.

**Table 4:** Descriptive statistics of the nine domains of quality of life 36-HAT-QoL, of patients seen at the HIV/AIDS outpatient clinic, Campinas (SP), 2017–2018.

Variables^a^		Women (140)	Men (205)	Total (345)	p-value
		f (%)	f (%)	f (%)	
General Function					
	Median (25.75)	90 (76.6–100)	100 (86.6–100)	93.33 (80–00)	0.00^†^
	Mean±SD	83.51 (9.11)	88.73 (18.18)	86.57 (18.72)	0.01^‡^
Life Satisfaction					
	Median (25.75)	80 (60–95)	85 (70–100)	85.00 (65–100)	0.003^†^
	Mean±SD	75.24 (20.71)	80.66 (20.39)	78.41 (20.66)	0.016^‡^
Health Concerns					
	Median (25.75)	95 (75–100)	100 (80–100)	95.00 (80–100)	0.044^†^
	Mean±SD	82.79 (22.42)	88.95 (16.72)	86.40 (19.49)	0.003^‡^
Financial Concerns					
	Median (25.75)	60 (40–93.3)	80 (40–100)	60.00 (40–100)	0.010^†^
	Mean±SD	59.95 (29.47)	67.93 (30.91)	64.62 (30.54)	0.016^‡^
Medication Concerns					
	Median (25.75)	96 (84–100)	100 (92–100)	96 (88–100)	0.002^†^
	Mean±SD	88.75 (15.26)	94.47 (9.38)	92.10 (12.47)	0.001^‡^
Acceptance of HIV					
	Median (25.75)	100 (80–100)	100 (100–100)	100 (100–100)	0.003^†^
	Mean±SD	84.13 (26.62)	91.70 (20.18)	88.57 (23.33)	----
Confidentiality Concerns					
	Median (25.75)	20 (20–52)	28 (20–72)	24.0 (20–68)	0.022^†^
	Mean±SD	38.92 (26.99)	46.65 (30.76)	43.45 (29.46)	0.015^‡^
Confidence in the professional					
	Median (25.75)	100 (93.3–100)	100 (93.3–100)	100 (98.3–100)	----
	Mean±SD	94.94 (12.27)	94.11 (12.86)	94.45 (12.61)	0.546^‡^
Sexual Function					
	Median (25.75)	100 (40–100)	100 (100–100)	100 (70.0–100)	----
	Mean±SD	73.12 (34.01)	89.17 (28.93)	83.17 (28.93)	0.001^‡^
Overall quality of life					
	Median (25.75)	76.5 (69.4–83.5)	82.4 (77–87.1)	80.58 (72.4– 86.5)	0.001^†^
	Mean±SD	75.18 (11.81)	81.27 (10.85)	78.85 (11.61)	0.001^‡^

HIV: Human immunodeficiency virus; SD: standard deviation.

* Non-normal distribution — Shapiro-Wilk (0.001),

p of discrete variables: Kruskal-Wallis^†^ and Student's *t* ^‡^. ----Unable to calculate.

The variables associated with each domain and overall QoL are presented in Table 5. In the first domain, *General Function,* we observed that age, income, and having suffered prejudice are associated with the domain. Young patients, with higher income and who have not suffered prejudice, have better rates in this domain.

**Table 5:** Factors associated with the domains of quality of life 36-HAT-QoL in Poisson-Tweedie regression analysis among HIV/AIDS patients, Hospital das Clínicas, Universidade Estadual de Campinas, Campinas (SP), 2018.

Domains	Variable	Crude analysis		Multivariate analysis		
		β	p-value	Adjusted β	95%CI:	p-value
General Function	Age	-0.002	0.060	-0.002	0.004 to 0.000	0.028
	Sex (woman)	-0.053	0.037			
	Income	0.000	0.003	0.001	-0.006 to −0.005	0.014
	Years of formal education	0.008	0.002			
	Hospitalizations (yes)	-0.091	0.042			
	Prejudice (yes)	-0.096	0.001	-0.096	-0.144 to −0.047	0001
Life Satisfaction	Sex (woman)	-0.065	0.029	-0.001	-0.003 to −0.046	0.001
	Income	-0.001	0.003	0.001	0.008 to 0.005	0.052
	Religion (yes)	-0.096	0.019	-0.095	-0.175 to −0.016	0.025
	Housing*	-0.082	0.001	-0.341	0.534 to −0.144	0.001
	Prejudice (yes)	-0.072	0.013			
	CD4 cell count	0.001	0.007	0.001	0.005 to 0.001	0.001
	Hospitalizations (yes)	-0.013	0.010	-0.096	-0.191 to −0.001	0.047
Health Concerns	Sex	0.063	0.013	0.081	0.027 to 0.137	0.003
	Housing*	0.050	0.002	-0.111	-0.201 to −0.021	0.016
	CD4 count	0.001	0.022	0.006	0.001 to 0.005	0.014
	Prejudice (yes)	0.075	0.001	-0.052	-0.106 to 0.001	0.054
	Hospitalizations (yes)	0.116	0.007	0.087	0.006 to 0.167	0.034
	3/1 therapy regime (no)	0.066	0.003	-0.050	-0.095 to −0.005	0.029
Financial Concerns	Sex (woman)	0.131	0.012	-0.104	-0.003 to 0.005	0.039
	Skin color	0.026	0.001	0.020	0.009 to 0.030	0.001
	Years of formal education	0.367	0.001	0.197	0.001 to 0.394	0.049
	Housing*	-0.324	0.001	-0.263	-0.429 to −0.098	0.002
	Work status^†^	0.224	0.001	0.206	0.111 to 0.301	0.001
	Prejudice (yes)	0.005	0.190	0.005	0.000 to 0.000	0.050
Medication Concerns	Sex (woman)	-0.051	0.001	-0.048	-0.078 to −0.018	0.002
	Prejudice (yes)	0.024	0.052			
	Poor adhesion (yes)	0.051	0.008	0.051	0.014 to 0.088	0.006
Acceptance of HIV	Age	0.001	0.248			
	Sex (woman)	-0.074	0.015			
	Years of formal education	0.010	0.001	-0.100	-0.193 to −0.007	0.036
	Prejudice due to sex	0.086	0.044	0.019	0.010 to 0.028	0.001
	Hospitalizations	-0.108	0.023	0.155	0.030 to 0.279	0.015
Confidentiality Concerns	Sex	-0.209	0.047	-0.198	-0.120 to −0.010	0.042
	Marital status	-0.216	0.047			
	Prejudice due to SO^‡^	-0.239	0.036	-0.313	-0.541 to −0.084	0.007
	Viral load value	-0.001	0.001	-0.001	-0.003 to −0.001	0.023
	Diagnosis time	-0.013	0.117	-0.033	-0.060 to −0.006	0.017
	Treatment time	0.003	0.051	0.047	0.020 to 0.074	0.001
Confidence in the professional	Years of formal education	-0.004	0.024			
	Disease stage	-0.062	0.001			
	Prejudice	-0.037	0.024	-0.071	-0.067 to -0.036	0.029
	Treatment time	0.003	0.013	-0.035	-0.036 to −0.004	0.001
	CD4 count	0.005	0.145	0.005	0.006 to 0.005	0.027
Sexual Function	Age	-0.004	0.050			
	Sex	-0.211	0.001			
	Level of education	0.019	0.001	0.214	0.120 to 0.309	0.001
	Income	0.328	0.032	0.016	0.005 to 0.027	0.005
	Sexual orientation ^§^	0.134	0.020			

HIV: Human immunodeficiency virus.

* Assigned dwelling or occupation;

^†^unemployed; ^‡^prejudice due to sexual orientation (SO); ^§^homosexual.

The second domain, *Life Satisfaction,* is associated with sex, income, religion, type of housing, CD4 cell count, and hospitalizations in the last year. Men, individuals with higher monthly income, who have some religion, have their own house, who were not hospitalized in the last year, and presented values of CD4 cell count above 200 cel./mm^3^ presented better satisfaction with life.

The third domain*, Health Concerns*, was associated with the variables sex, type of housing, prejudice, hospitalizations, and therapy regime TDF+3TC+EFZ (3/1). Women, individuals who live in assigned or occupied dwellings, who suffered some prejudice, with CD4 cell count less than 200, who were hospitalized in the last year, and are not treated with TDF+3TC+EFZ have greater concern for their health.

In the fourth domain, *Financial Concerns*, we found a mean (SD), marking a moderate quality of life. This domain was associated with sex, level of education, type of housing, work status, prejudice, and CD4 cell count. Women, patients with low level of education, unemployed, without their own house, who have suffered prejudice especially due to race, and whose CD4 cell count is below 200 have greater concern in this regard.

*Medication Concerns* are evaluated in the fifth domain. The four items were answered by 346 patients who used antiretroviral drugs; the four individuals who did not receive medication were patients named as Elite Controllers. Only the variables sex and adherence to treatment were associated: women and patients with poor adherence have greater concern about the effects of drugs.

The *Acceptance of HIV* was evaluated in the sixth domain, having as associated factors sex, level of education, and prejudice due to sex. Women, patients with low levels of education, and those who suffered prejudice have lower levels of disease acceptance.

The mean (SD) of the seventh domain, which deals with the disclosure of the confidentiality about HIV infection (*Confidentiality Concerns*), scored 43.45 (29.46), being considered low. Prejudice due to sexual orientation, diagnosis time, and treatment time were associated with sex. Women, homosexuals, those who had less time of diagnosis and treatment were more afraid that the diagnosis of HIV was known to others.

The eighth domain, *Confidence in the professional*, had a higher mean. The description of this domain, on the original scale, shows that it can be used to evaluate any professional of the healthcare team. In this study, we chose to evaluate the relationship with the physician because the medical appointment is the most regular and systematic in outpatient follow-up. The stage of the disease (CDC-B and C), prejudice, and treatment time are associated with this domain.

The ninth domain, *Sexual Function*, obtained a mean index (SD) of 83.1 points (20.0–100.0). According to the results of the multivariate analysis, we verified an association of the variables women and low level of education with the low score.

## DISCUSSION

We verified that the QoL domains most affected in the evaluated population were LS, FC, and CC, as described in other studies^12,13^. These domains were associated, respectively, with the following variables: sociodemographic (women, income, housing) and clinical (CD4 cell count and hospitalizations); sociodemographic (race and work status) and having suffered prejudice; and confidentiality concerns, sex, prejudice, and clinical variables (CD4 cell count and times of diagnosis and treatment).

Although the objective of the study was to evaluate the differences in QoL of HIV/AIDS patients by self-reported race/skin color, by conducting the research we were able to verify that Black participants had lower level of education and income, are in a higher proportion unemployed, and live in rented or assigned houses when compared to white individuals. However, the differences in QoL are marked by sex, income, and level of education, in accordance with what has been described by several authors, who claim that in Brazil the epidemic spread among residents of poorer communities, especially among women^14^, those with fewer years of formal education^10^, and among those who have poorly-paid occupations or who are excluded from the formal labor market^10,15^. That is, progressively, the epidemic began to reflect the prevailing patterns of inequality in the country^16^.

The findings corroborate that gender inequalities are one of the main underlying structural drivers of health equity, as described by the Commission on Health Equity and Inequalities in the Americas^17^. In the research, both income and level of education and the levels of several QoL domains have lower values among women when compared with men. Regarding the QoL indexes, our data are similar to the study conducted among women with HIV, which indicates impairment in the domains FC, HC, CC, AND SF^13^; however, no association was found with LS, AHIV, and MC, as in the present study. Nonetheless, authors of a study carried out in a municipal hospital in the state of Rio Grande do Norte, Brazil, when evaluating this variable (sex), found differences in the domains FC, MC, and SF^12^ among women.

However, Black patients had low levels of QoL only in the FC domain; and it is worth noting the difficulties that this group presents in access to diagnosis and treatment, because these individuals access it mainly by the flows of the public health network, but especially by the emergency care services, ending up having worse health conditions than white patients. Delay in access to the specialized service and in treatment onset enables clinical complications and a higher risk of mortality, as described in other studies^18,19^. Furthermore, it is observed that the proportion of patients with CDC-C clinical condition (AIDS) among this group (32.9%), hospitalizations in the last year (11.1%) and their cause (HIV) (38.3%) is higher among those with mixed-race/Black skin color when compared to those with white skin color. Social determination is marked in the course followed by Black patients to receive adequate treatment and have adequate QoL, especially being HIV carriers.

As stated by Albuquerque et al., racial inequality has been a factor of influence and/or determination of the place of individuals in society, defining their access, greater or lesser, to wealth, education, housing, public assets, healthcare services, information, among others^20^. In this study, we can perceive these inequalities particularly in the Black and female population.

According to Galvão et al., the literature highlights how racism operates not only explicitly, but also in subliminal mechanisms that generate cumulative and lasting effects^21^. Structural racism, solidified and internalized in the culture of a people, excludes a portion of the population from access to education, employment, upward social mobility, information and, hence, to health. Conversely, institutional racism results from policies, practices, and procedures of institutions that have a negative effect on access of racial minorities as well as on the quality of assets, services, and opportunities^22^. Although science has advanced knowledge of health inequalities, experiences of racism are still underestimated.

Corroborating our findings, the QoL of PLHIV is not only related to the possibility of a longer life, because coping with the disease becomes increasingly problematic, especially when it comes to the lack of social and financial resources^16^. In the FC domain, we investigated the concern of having to live on a fixed income, having to pay the bills, or even simply having or not the opportunity to have leisure or some satisfaction with their monthly income^23^.

Individuals who live in poverty experience serious difficulties in accessing treatment, which may cause harm to live with HIV^24^. In our study, we found that the sample participants are in a situation of financial limitation, with those who are Black and women at a higher level, reinforcing socioeconomic differences.

The MC domain investigates whether the fact that patients have to take drugs makes them feel more sick or healthier when they realize they are fighting the virus. It also investigates whether drugs interfere with normal life (side effects)^25^. Inadequate adherence to treatment with antiretroviral drugs is, in some cases, associated with the possibility of disclosing the serological condition by the use of medicines, as stated by authors of a study conducted in Thailand^26^. In the present study, we observed an association between the MC and CC domains.

The domain that deals with the disclosure of the confidentiality of HIV infection is evaluated as the concern and/or limitation regarding the disclosure of the infection diagnosis to family members and coworkers, as well as the concern that people discover their diagnosis^27^. In the evaluated sample, 50% of the patients reported that "nobody knows about the disease," which justifies the low score presented, but it is shown as a strategy to protect themselves from the prejudices that still exist in our society. Logie et al.^28^ state that HIV-related stigma and racial and gender discrimination are correlated and compromise the physical and mental health and the QoL of this group, as described in the present study.

As in other studies, we verified that confidence in the professional is more focused on the figure of the physician. According to research, the figure of the medical professional may lead the patient to remain well physically, to develop the various activities suggested, and to have better QoL^13^. However, Guibu et al.^29^ show that access to the multiprofessional team is related to greater survival of AIDS patients in the South and Southeast regions of Brazil.

According to studies, the interference in QoL of HIV patients is multifactorial. Confidentiality about the disease, socioeconomic conditions — such as work and income —, emotional stress — intensified by discrimination and poverty — negatively impact different spheres of the life of individuals, which coincides with our findings^24,25^.

It should be noted that, among the study limitations, we mention its cross-sectional design, which prevents the identification of causality; the sample by weekly systematic recruitment, and the use of self-reported variables, which may compromise representativeness by presenting selection and information biases. Moreover, the absence of control for contextual variables and the collection restricted to a single outpatient clinic limit the reach of the findings and their generalization.

The inequalities observed in people living with HIV/AIDS in this study are similar to those found worldwide. We can conclude that, even in the face of well-defined public policies of comprehensive health care, which reinforce universal access to antiretroviral therapy^1,30^, there are still differences in access to treatment and in QoL by sex and skin color in this group.

## Supplementary Material



## Footnotes

1 The Hospital Dia was chosen because it is linked to the Hospital das Clínicas of Unicamp and serves patients living with HIV/AIDS and infectious diseases, being a reference in the region.

2 Poor adherence was considered as the discontinuity of follow-up by two appointments and the non-withdrawal of medicines in the pharmacy.
