# Supplementary material for: Evaluation of sociodemographic, clinical and behavioral characteristics of people living with the human immunodeficiency virus and its association with quality of life
Source: Rev Bras Epidemiol. 2025 Jun 27;28:e250034. doi: 10.1590/1980-549720250034 (PMC12204236; doi:10.1590/1980-549720250034)
Supplement: Supplementary file 1 [file 1980-5497-rbepid-28-e250034-suppl1.pdf]

Apêndice 1. Correlações de Pearson entre as escalas do HIV/AIDS-Targeted Quality of Life Instrument em português brasileiro e as características sociodemográficas e de gravidade da doença

	HIV/AIDS-Targeted Quality of Life Instrument[a]								
	**FG**	**SV**	**PS**	**PF**	**PM**	**AHIV**	**PSi**	**CP**	**FS**
Idade	-0,092	0,005	0,096	0,021	-0,045	0,049	0,034	0,084	**-0,116**[b]
Sexo	**0,145**[c]	**0,115**[b]	**0,189**[c]	**0,117**[b]	**0,186**[c]	**0,177**[c]	0,104	-0,027	**0,200**[c]
Cor da Pele	-0,008	0,044	-0,045	**-0,175**[c]	0,030	-0,055	0,019	0,061	-0,032
Estado Civil	0,005	-0,088	-0,072	0,022	0,076	0,017	**0,137**[b]	-0,085	-0,005
Escolaridade	**0,166**[c]	0,080	0,061	**0,206**[c]	**0,109**[b]	**0,152**[c]	0,011	**-0,122**[b]	**0,237**[c]
Renda Percapita	**-0,160**[c]	**0,117**[b]	0,076	**0,327**[c]	-0,040	0,106	0,074	-0,100	**0,149**[c]
Situação Laboral	-0,091	-0,039	-0,046	-0,078	0,016	0,027	0,066	0,034	-0,013
Orientação Sexual	**0,126**[b]	0,000	-0,004	0,086	0,105	0,052	0,058	-0,180[c]	**0,143**[b]
Sofrer Preconceito	**0,213**[c]	**0,145**[c]	**0,123**[b]	**0,209**[c]	**0,133**[b]	0,103	0,008	**0,134**[b]	0,062
Tempo Soropositividade	0,017	-0,034	0,058	-0,100	0,047	0,046	-0,052	0,039	0,045
Contagem CD4	0,046	**0,150**[c]	**0,133**[b]	0,058	0,022	0,078	-0,042	0,089	-0,012
Condição Clínica	-0,068	0,013	-0,045	-0,020	0,060	-0,011	**0,109**[b]	**0,223**[c]	-0,009
Internações	**0,155**[c]	**0,154**[c]	**0,170**[c]	0,004	0,085	**0,153**[c]	0,024	-0,011	0,002

[a]FG: Função Geral, SV: Satisfação com a Vida; PS: Precauções com a Saúde; PF: Precauções Financeiras; PM: Precauções com os medicamentos; AHIV: Aceitação do HIV; PSi: Precaução com o Sigilo; CP: Confiança no Profissional e FS: Função Sexual. Altamente correlacionados: ≥ 0,75; moderadamente correlacionados: 0,51 a 0,75; pouco correlacionados:0,26 a 0,50; minimamente correlacionados: ≤ 0,25.

[b] $p < 0,05$;

[c] $p < 0,01$.

Apêndice 2. Características sociodemográficas de pacientes atendidos no ambulatório de HIV/Aids em relação ao sexo, Campinas, SP - 2018-2021

Variáveis	Mulher (140)	Homem (205)	Total (345)	*p*
	f (%)	f (%)	f (%)	
**Idade**				
Mediana (25,75)	46 (37, 54)	43 (36, 50)	45 (37, 53)	0,720
média ± DP	45,79 (12,4)	43,7 (12,2)	45,2 (12,6)	
Mínimo - Máximo	18 - 76	18 - 76	18 - 76	
**Estado Civil**				
Solteiro/Separado	75 (21,7)	136 (39,4) 69	211 (61,2)	0,131
Casado/União Livre	65 (18,8)	(20,0)	134 (38,8)	
**Escolaridade**				
Mediana (25,75)	9 (4; 10,5)	9 (4, 10)	9 (4. 12)	**0,052**
média ± DP	8,76 (4,04)	9,96 (4,37)	9,29 (4,24)	
Mínimo - Máximo	4 - 19	0 - 19	0 - 19	
**Escolaridade**				
Analfabeto	0 (0,0)	2 (0,6)	2 (0,6)	
Ensino Fundamental	67 (19,6)	76 (22,3)	143 (41,9)	0,129
Ensino Médio	37 (10,9)	59 (17,3)	96 (28,2)	
Superior	37 (10,0)	66 (19,4)	100 (29,3)	
**Renda Percapita**				
Mediana (25,75)	500(300; 900)	900(450;1667)	666(357;1400)	**0,009**
Media ± DP	891 (1515)	1387 (1491)	1181 (1519)	
Mínimo - Máximo	0 - 11667	0 - 10000	0 - 11667	
**Religião**				
Sim	117 (33,9)	154 (44,6)	271(78,6)	0,060
Não	23 (6,7)	51 (14,8)	74 (21,4)	
**Tipo de Religião**				
Católico	66(24,3)	82 (30,1)	148 (54,4)	
Evangélico	34 (12,5)	41 (15,1)	75 (27,6)	0,093
Espírita	6 (2,2)	22 (8,1)	28 (10,3)	
Outros	11 (4,0)	10 (3,7)	21 (7,7)	
**Situação Trabalho**				
Afastado	2(6,0)	1 (5,7)	41 (11,7)	
Aposentado	31 (9,8)	43 (12,5)	36 (10,2)	
Empregado	52 (15,1)	93 (27,0)	146 (41,7)	0,505
Desempregado	33 (9,6)	38 (11,0)	75 (21,4)	
Outros (Estudantes)	22 (6,4)	30 (8,7)	52 (14,9)	
**Condição habitação**				
Própria	92 (26,7)	128 (37,1)	220 (63,8)	
Alugada	36 (10,4)	53 (15,4)	89 (25,8)	0,091
Cedida	12 (3,5)	15 (4,3)	27 (7,8)	
Outras (Ocupação)	0 (0,0)	9 (2,6)	9 (2,6)	
