# Supplementary material for: Evaluation of sociodemographic, clinical and behavioral characteristics of people living with the human immunodeficiency virus and its association with quality of life
Source: Rev Bras Epidemiol. 2025 Jun 27;28:e250034. doi: 10.1590/1980-549720250034 (PMC12204236; doi:10.1590/1980-549720250034)
Supplement: Translation

# Translation: Avaliação das caraterísticas sociodemográficas, clínicas e comportamentais de pessoas que vivem com o vírus da imunodeficiência humana (HIV/AIDS) e sua associação com a qualidade de vida

**DOI:** 10.1590/1980-549720250034.2

**Authors:** Ehideé Gómez La-Rotta, Leidy Janeth Erazo Chavez, Harold Gomez-Larrota, Pedro Henrique de Faria, Armindo Augusto da Nobrega Albuquerque, Felipe Thiele Cecílio, Max da Silva Maciel, José Antonio Enciso Domínguez, Maria Rita Donalisio

**Affiliations:** I Universidade da Integração Latino-Americana, Curso Saúde Coletiva – Foz do Iguaçu (PR), Brasil.; II Universidade Federal do Maranhão, Programa de Pós-Graduação em Saúde Coletiva – São Luís (MA), Brasil.; III Universidade Ceuma, Curso de Medicina e Psicologia – São Luís (MA), Brasil.; IV NTS Electronic Control – Bogotá, Cundinamarca, Colômbia.; V Universidade Estadual de Campinas, Faculdade de Ciências Médicas, Pós-Graduação em Saúde Coletiva – Campinas (SP), Brasil.; VI Universidade de São Paulo, Hospital de Clínicas – São Paulo (SP), Brasil.

**Keywords:** Qualidade de vida, Sobreviventes de longo prazo ao HIV, Fatores raciais, Sexo, Desigualdades

## Abstract

**Objetivo:**

Investigar desigualdades de raça/cor da pele e sexo com relação à qualidade de vida de pessoas que vivem com o vírus da imunodeficiência humana (HIV) em um hospital universitário brasileiro entre 2017 e 2018.

**Métodos:**

Realizamos estudo transversal entre 2017 e 2018 com 350 pessoas que vivem com o HIV, sendo aplicada a escala de qualidade de vida (QV) específica para HIV. Os grupos foram comparados com os testes de χ^2^ e teste *t* de Student ou Kruskal-Wallis. Para avaliar os fatores associados aos nove domínios da QV, realizou-se regressão de Tweedie; uma aplicação do modelo linear generalizado.

**Resultados:**

Dos 350 participantes, 55,7% foram autorreferidos de cor da pele branca e 44,3% de cor da pele parda/preta; com média de idade (desvio padrão — DP) de 45,2 (±12,6). Constatou-se que 46,3% referiram ter sofrido algum tipo de preconceito, 34,8% pela doença, 12% pela cor da pele e 11,4% pela orientação sexual. A média geral qualidade de vida foi de 78,85 (±11,61). O domínio com menor média foram preocupações com o sigilo (M: 43,45±29,46). Entre os fatores associados a vários domínios encontramos sexo (feminino), escolaridade, renda *per capita*, ter sofrido algum preconceito ou internações no último ano.

**Conclusão:**

Destacaram-se diferenças na escolaridade, renda *per capita* e situação de trabalho por raça/cor autorreferida dos participantes, mostrando desigualdades na população de estudo. O sexo (feminino) é o fator que se associa à maioria dos domínios da QV, entre eles satisfação com a vida, preocupação pelo sigilo, saúde, situação financeira e medicamentos, aceitação da infecção por HIV e atividade sexual.

## INTRODUÇÃO

Em 2023, 39 milhões de pessoas no mundo viviam com HIV/Aids, número que segue aumentando em razão do incremento da sobrevida pelo acesso ao tratamento antirretroviral (TARV)^1^.

Na América Latina, o número de casos novos de HIV em 2022 foi de 110 mil, embora o número de mortes tenha diminuído^1,2^. No Brasil, foram notificados 43.403 casos de infecção pelo HIV, sendo diagnosticados 6.759 (44,6%) no estado de São Paulo. Entretanto, a taxa de mortalidade no Brasil sofreu decréscimo de 26,5% entre 2012 e 2022^3^.

Embora no Brasil exista um programa nacional bem-organizado para combater a AIDS, deve-se considerar que os aspectos econômicos e políticos têm um significado essencial para cada população^4^. A maioria dos casos está localizada em países pobres, sugerindo a necessidade de analisar os determinantes sociais e desigualdades nessas populações^5^.

Nesse sentido, observa-se que no Brasil houve aumento da incidência de casos de HIV nas populações vulneráveis e de menor nível socioeconômico, entre eles os de cor da pele parda/preta, acentuando o racismo estrutural e as condições de vulnerabilidade dessa população^3,6^. A proporção de infeções pelo HIV entre pessoas de cor da pele parda/preta foi de 60,1% (48,4% de cor da pele parda e 11,7% de cor da pele preta), e nos de cor da pele branca de 33,1% em 2022; observando-se que os óbitos se deram em 61,7% (47,0% em cor da pele parda e 14,7% em cor da pele preta) e 35,6% na população de cor da pele branca^3^.

Com o aumento das taxas de sobrevivência de pessoas com HIV/AIDS, o que a caracteriza como uma doença crônica, a assistência à saúde prestada a esse grupo ganha maior importância pela necessidade de cuidados únicos para manter sua qualidade de vida (QV)^4^.

O objetivo foi investigar desigualdades de raça/cor da pele e sexo com relação à QV de pessoas que vivem com HIV em um hospital universitário brasileiro.

## MÉTODOS

Realizamos estudo transversal com pessoas que vivem com HIV (PVHIV) autorreferidas de cor da pele parda/preta e de cor da pele branca acompanhadas nos ambulatórios de infectologia do Hospital das Clínicas da Universidade Estadual de Campinas (HC-Unicamp)^1^ entre outubro de 2017 e abril de 2018. A pesquisa foi aprovada pelo Comitê de Ética em Pesquisa (CEP), com parecer 2.148.789, de agosto de 2017.

Com taxa de detecção de HIV em 2019 de 17,8 casos por 100 mil habitantes no Brasil, calculou-se o número mínimo de indivíduos. Dada a população de 4.900 PVHIV em seguimento no ano de 2015, com máximo erro amostral 5% e intervalo de confiança de 95%, calculou-se uma amostra mínima de 357 indivíduos.

O recrutamento dos pacientes foi realizado por captação sistemática semanalmente e contato direto, durante o comparecimento em consulta, ao longo de oito meses de coleta de dados, com seleção de acordo com os seguintes critérios: pacientes HIV positivos, maiores de 18 anos, acompanhados nos ambulatórios de infectologia e que não sofressem de algum déficit cognitivo que os impossibilitasse de seguir as orientações.

As entrevistas foram realizadas face a face no dia da consulta do paciente, em local reservado. Posteriormente, foram analisados os prontuários deles para obter informações sobre variáveis clínicas (classificação da condição clínica, valores de CD4, carga viral, internações no último ano, causas das internações, adesão ao tratamento^2^, medicamentos e doses) no dia da entrevista ou meses depois, respeitando-se o que foi aprovado e pleiteado no CEP.

As informações foram coletadas por meio do preenchimento de questionário físico, com cinco seções que investigavam características sociodemográficas (cor da pele referida, idade, sexo, estado civil, escolaridade, renda *per capita* (salário-mínimo — R$ 937; 293US$ para 2018), religião, situação laboral, orientação sexual, se sofre preconceito (em razão de cor da pele, sexo, ser portador de HIV/Aids, orientação sexual), comportamento de risco para a infecção por HIV, evolução clínica e gravidade da doença.

Para avaliar a QV, foi utilizado o questionário Quality of Life (HAT-QoL)^7,8^, validado para o português brasileiro^9^ e composto de 34 itens que avaliam nove domínios de QV: função geral (FG), satisfação com a vida (SV), preocupação com a saúde (PS), preocupações financeiras (PF), preocupações com a medicação (PM); aceitação do HIV (AHIV), preocupações com o sigilo (PSi), confiança no profissional (CP) e atividade sexual (AS).

Para responder a cada questão, o indivíduo era conduzido a pensar sobre a sua qualidade de vida nas últimas quatro semanas. As respostas tinham formato de escala do tipo Likert de cinco pontos. Em cada domínio, 0 é o escore mais baixo e 100 é o melhor escore possível. Quanto maior o escore, menor o impacto da infecção pelo HIV na QV dos indivíduos, e vice-versa^9^.

O banco de dados foi alimentado em planilha Excel por meio de recursos do Google. Posteriormente foi transferido e analisado utilizando o programa Statistical Package for the Social Sciences (SPSS), versão 21.0. (SPSS, Chicago, IL).

Calculamos as proporções das variáveis categóricas e as medidas de tendência central e de dispersão para as variáveis contínuas. As variáveis categóricas foram comparadas com os testes de χ^2^ e exato de Fisher, as médias com o teste *t* de Student para amostras independentes; e as medianas com Kruskal-Wallis. O valor de p<0,05 foi considerado como marcador de diferença estatística em todas as análises.

As variáveis de desfecho foram os domínios da QV e as variáveis de exposição foram cor da pele autorreferida e sexo, além de outras variáveis sociodemográficas, clínicas, acesso e preconceito. Para determinar a correlação entre as variáveis sociodemográficas e clínicas e os domínios de QV avaliados com o HAT-QoL foi usado o método de Pearson^10^. Entretanto, os resultados encontrados nesta pesquisa mostram pouca correlação entre as variáveis sociodemográficas e clínicas e os domínios de QV (Apêndice 1).

Para avaliar a associação dos domínios da QV com as covariáveis coletadas (variáveis independentes), realizamos regressão de Tweedie, uma aplicação do modelo linear generalizado. Iniciamos pela análise univariada, que permitiu selecionar as variáveis que apresentaram valor de p<0,20 para a construção do modelo múltiplo. As variáveis que permaneceram no modelo múltiplo final foram as que apresentaram significância estatística (p<0,05) em procedimento *backwards*.

Nas Tabelas 1 e 2 foram agrupadas as populações de cor da pele parda/preta conforme é preconizado pelo Instituto Brasileiro de Geografia e Estatística (IBGE)^11^; e os de cor da pele branca e amarela foram agrupados na outra categoria.

**Tabela 1:** Características sociodemográficas e comportamentais de pacientes atendidos no ambulatório de HIV/Aids com relação à raça/cor da pele referida, Campinas (SP), 2017–2018.

Variáveis		Cor da pele branca (155)	Cor da pele parda/preta (195)	Total (350)	Valor-p
		f (%)	f (%)	f (%)	
Idade					
	Mediana (25,75)	48 (39,55)	43 (36,50)	45 (37,53)	0,013^§^
	Média±DP	47,12 (12,9)	43,7 (12,2)	45,2 (12,6)	
	Mínimo–Máximo	20–6	18–76	18–76	
Sexo					
	Homem	97 (62,6)	108 (55,4)	205 (58,6)	0,262^‡^
	Mulher	55 (35,5)	85 (43,6)	140 (40,0)	
	Transgênero; Outro	3 (1,9)	2 (1,0)	5 (1,4)	
Estado civil					
	Solteiro/Separado/Viúvo	100 (64,5)	116 (59,5)	216 (61,7)	0,376^†^
	Casado/União livre	55 (35,5)	79 (40,5)	134 (38,3)	
Escolaridade					
	Mediana (25,75)	10 (8,15)	9 (4,10)	9 (4,12)	0,013^§^
	Média±DP	10,2 (4,56)	8,56 (3,84)	9,29 (4,24)	
	Mínimo–Máximo	4–19	0–19	0–19	
Renda *per capita* *					
	Mediana (25,75)	937 (465,2000)	505 (312,1000)	666 (357,1400)	0,001^//^
	Média±DP	1.506 (1602)	927 (1403)	1.181 (1519)	
	Mínimo–Máximo	0–10.000	0–11.666	0–11.666	
Orientação sexual					
	Heterossexual	98 (63,2)	159 (81,5)	257 (73,4)	0,001^†^
	Homossexual	47 (30,3)	29 (8,3)	76 (21,7)	
	Outro (Bissexual)	10 (6,5)	7 (2,0)	17 (4,9)	
Número parceiros*					
	Mediana (25,75)	1 (1,1)	1 (1,1)	1 (1,1)	0,754^//^
	Média±DP	1,92 (4,73)	1,57 (2,84)	1,73 (3,80)	
	Mínimo–Máximo	0–50	0–30	0–50	
Uso de camisinha*					
	Sim	103 (65,5)	149 (76,4)	252 (72,0)	0,002^†^
	Não	23 (14,8)	18 (9,2)	41 (11,7)	
	Não se aplica	29 (18,7)	28 (14,4)	57 (16,3)	
Drogas recreativas					
	Sim	79 (51,0)	85 (43,6)	164 (46,9)	0,196^†^
	Não	76 (49,0)	110 (56,4)	186 (53,1)	
Tipo de drogas^¶^					
	Álcool (sim)	52 (34,4)	57 (30,5)	109 (32,2)	0,512^†^
	Fumo (sim)	34 (23,8)	48 (25,8)	82 (24,9)	0,673^†^
	Maconha (sim)	10 (6,6)	2 (1,1)	12 (3,5)	0,006^‡^
	*Crack* (sim)	1 (0,7)	5 (2,6)	6 (1,8)	0,334^‡^
	Cocaína (sim)	1 (0,7)	2 (1,0)	3 (0,9)	0,539^‡^
	Outras (sim)	2 (1,3)	0 (0,0)	2 (0,6)	0,069^‡^

HIV: Vírus da imunodeficiência humana; DP: desvio padrão.

* Distribuição não normal — Shapiro-Wilk (0,001);

p das categóricas: testes de χ^2†^ e exato de Fisher^‡^. p das discretas: t de Student^§^ e Kruskal-Wallis^//^; ^¶^Apresentam-se os dados dos que estão consumindo no momento.

**Tabela 2:** Acesso e aspectos clínicos de pacientes atendidos no ambulatório de HIV/Aids com relação à raça/cor da pele referida, Campinas (SP), 2017–2018.

Variáveis		Cor da pele branca (155)	Cor da pele parda/preta (195)	Total (350)	Valor-p
		f (%)	f (%)	f (%)	
Forma de acesso					
	PS/Emergência	45 (31,5)	70 (37,2)	115 (34,7)	0,007^‡^
	SUS/UBS	35 (24,5)	61 (32,4)	84 (29,0)	
	Médico/Convênio	31 (21,7)	14 (7,4)	45 (13,6)	
	CAISM	14 (9,8)	16 (8,5)	30 (9,1)	
	Busca espontânea	6 (4,2)	11 (5,9)	17 (5,1)	
	Hemocentro	4 (2,8)	10 (5,3)	14 (4,2)	
	Outro	8 (5,6)	6 (3,2)	14 (4,2)	
Tempo diagnóstico*					
	Mediana (25,75)	13 (6,4-20,3)	12,3 (5,3;19)	12,3 (5,9;19,3)	0,209^//^
	Média±DP	13,4 (8,02)	12,3 (12,2)	12,78 (7,9)	
	Mínimo–Máximo	0,59–34,2	0,43–33,3	0,43–34,2	
Tempo tratamento*					
	Mediana (25,75)	11,1 (5,1–19,4)	10,7 (4,2–18)	10,9 (4,8–18,1)	0,354^//^
	Média±DP	11,9 (7,72)	11,2 (7,56)	11,5 (7,63)	
	Mínimo–Máximo	0,20–27,5	0,11–28,0	0,11–28,0	
Diferença entre Dx e início tratamento*					
	Mediana (25,75)	0,08 (0,01; 1)	0,08 (0,01;0,5)	0,08 (0,0–0,7)	0,482^//^
	Média±DP	1,59 (3,63)	1,14 (2,76)	1,34 (3,18)	
	Mínimo–Máximo	3,33–19	0,01–16	3,33–19	
Condição clínica^¶^					
	CDC A	68 (43,9)	74 (37,9)	143 (40,6)	0,230^†^
	CDC B	31 (20,0)	32 (16,4)	63 (18,0)	
	CDC C	56 (36,1)	89 (45,6)	145 (41,4)	
CD4 células/mm^3^					
	<200	9 (5,8)	15 (7,8)	24 (6.9)	0.771^†^
	200–499	51 (33,1)	63 (32,8)	114 (32.6)	
	>500	94 (61,0)	114 (59,4)	208 (60,1)	
Carga viral^#^					
	<50	138 (90,2)	167 (86,1)	305 (87.9)	0,442^†^
	51–10.000	10 (6,5)	20 (10,3)	30 (8.6)	
	>10.001	5 (3,3)	7 (3,6)	12 (3,5)	
Tratamento					
	Sim	153 (98,7)	193 (99,0)	346 (98,9)	0,817^†^
	Não	2 (1,3)	2 (1,0)	4 (1,1)	
Número de medicamentos					
	Mediana (25,75)	3 (1,0;5,0)	3 (1,0;3,0)	3 (1,0;4,0)	0,229^§^
	Média±DP	3,03 (2,23)	2,78 (1,82)	2,89 (2,01)	
	Mínimo	0	0	0	
	Máximo	10	10	10	
Má adesão					
	Sim	40 (25,8)	56 (28,7)	96 (27,4)	0,544^†^
	Não	115 (74,2)	139 (71,3)	254 (72,6)	
Internações último ano					
	Sim	22 (14,2)	39 (20,0)	61 (17,4)	0,155^†^
	Não	133 (85,8)	158 (80,0)	289 (82,6)	
Causa internações					
	Pelo HIV	11 (50,0)	24 (61,5)	35 (57,4)	0,382^†^
	Por outras causas	11 (50,0)	15 (38,5)	26 (42,6)	

HIV: Vírus da imunodeficiência humana; PS: Pronto-Socorro; SUS: Sistema Único de Saúde; UBS; Unidade Básica de Saúde; CAISM: Centro de Atenção Integral à Saúde; DP: desvio padrão; Dx: Diagnóstico; CDC: Centers for Disease Control and Prevention; CDC A: doença não avançada; CDC B: doença moderadamente avançada; CDC C: doença avançada.

* Distribuição não normal — Shapiro-Wilk (0,001), medida em anos;

p das categóricas: testes de χ^2†^ e exato de Fisher^‡^; p das discretas: t de Student^§^ e Kruskal-Wallis^//^; ^¶^Conforme critérios dos Centers for Disease Control and Prevention (CDC); ^#^Cópias/mL^3^.

## RESULTADOS

### Características socioeconômicas e demográficas

Foram entrevistados 350 pacientes, com média de idade (desvio padrão — DP) de 45,2 (±12,6); 58,6% eram do sexo masculino, 33,8% eram casados/união livre e 42,2% referiam ensino fundamental, com média de 9,3 (±4,2) anos de escolaridade. Foram 195 (55,7%) pessoas de cor da pele branca e 155 (44,3%) cor da pele parda/preta com diferenças na escolaridade, na renda *per capita* e na situação de trabalho (p<0,001). Os pacientes que referiram ser de cor da pele parda/preta têm baixos níveis de escolaridade, baixa renda e situações de trabalho diferentes (Tabela 1).

Entre os sexos, encontraram-se diferenças: os homens tinham médias de escolaridade maiores e contavam com melhor renda (Tabela 3).

**Tabela 3:** Características sociodemográficas e comportamentais de pacientes atendidos no ambulatório de HIV/Aids com relação ao sexo, Campinas (SP), 2017–2018.

Variáveis		Mulher (140)	Homem (205)	Total (345)	Valor-p
		f (%)	f (%)	f (%)	
Idade					
	Mediana (25,75)	46 (37,54)	43 (36,50)	45 (37,53)	0,720^§^
	Média±DP	45,79 (12,4)	43,7 (12,2)	45,2 (12,6)	
	Mínimo–Máximo	18–76	18–76	18–76	
Estado civil					
	Solteiro/Separado	80 (55,2)	136 (66,3)	211 (61,7)	0,131^†^
	Casado/União livre	65 (44,8)	69 (33,7)	134 (38,3)	
Escolaridade					
	Mediana (25,75)	9 (4; 10,5)	9 (4; 10)	9 (4; 12)	0,052^§^
	Média±DP	8,76 (4,04)	9,96 (4,37)	9,29 (4,24)	
	Mínimo–Máximo	4–19	0–19	0–19	
Renda *per capita*					
	Mediana (25,75)	500 (300; 900)	900 (450; 1667)	666 (357; 1400)	0,009^//^
	Media±DP	891 (1515)	1.387 (1491)	1.181 (1519)	
	Mínimo–Máximo	0–11667	0–10000	0–11667	
Orientação sexual					
	Heterossexual	139 (95,8)	118 (57,6)	257(73,4)	0,001^†^
	Homossexual	3 (2,1)	73 (35,6)	76 (21,7)	
	Outro (Bissexual)	3 (2,1)	14 (6,8)	17 (4,9)	
Número parceiros*					
	Mediana (25,75)	1 (0,1)	1 (1,2)	1 (1,1)	0,001^//^
	Média±DP	0,74 (0,75)	2,42 (4,81)	1,73 (3,80)	
	Mínimo–Máximo	0–7	0–50	0–50	
Uso de camisinha*					
	Sim	87 (60,0)	165 (80,5)	252 (72,0)	0,001^†^
	Não	14 (9,7)	27 (13,2)	41 (11,7)	
	Não se aplica	44 (30,3)	13 (6,3)	57 (16,3)	
Drogas recreativas					
	Sim	60 (41,4)	104 (50,7)	164 (46,8)	0,084^†^
	Não	85 (58,6)	101 (49,3)	186 (53,1)	
Tipo de drogas^¶^					
	Álcool (sim)	35 (24,5)	74 (37,9)	109 (32,2)	0,009^†^
	Fumo (sim)	30 (21,6)	52 (27,4)	82 (24,9)	0,231^†^
	Maconha (sim)	3 (2,1)	9 (4,6)	12 (3,5)	0,212^‡^
	*Crack* (sim)	2 (1,4)	4 (2,0)	6 (1,8)	0,662^‡^
	Cocaína (sim)	1 (0,7)	2 (1,0)	3 (0,9)	0,757^‡^
	Outras (sim)	0 (0,0)	2 (1,0)	2 (0,6)	0,228^‡^

HIV: Vírus da imunodeficiência humana; DP: desvio padrão.

* Distribuição não normal — Shapiro-Wilk (0,001);

p das categóricas: testes de χ^2†^ e exato de Fisher^‡^; p das discretas: t de Student^§^ e Kruskal-Wallis^//^; ^¶^Apresentam-se os dados dos que estão consumindo no momento.

### Características comportamentais e epidemiológicas

Como descrito na Tabela 1, 73,4% referiram ser heterossexuais e 59,1% ter parceiro fixo, com média de 1,7 (±3,8) parceiro ao ano. Encontraram-se diferenças no número de parceiros no último ano e no uso de preservativo como meio de prevenção da infecção entre os sexos (p<0,001). Os homens tiveram média de 2,4 (±4,8), enquanto as mulheres tiveram média (DP) de 0,7 (±0,73) parceiro no último ano, e utilizaram com maior frequência (80,5%) o preservativo que as mulheres (60,0%).

No que se refere ao uso de drogas recreacionais, observamos que 164 (46,7%) as utilizam, sendo mais prevalente o uso do álcool — 109 (32,2%), tabaco — 82 (24,9%) e maconha — 12 (3,5%). Todas são consumidas com maior frequência pelos homens (p<0,001).

### Acesso ao serviço e características clínicas

Na Tabela 2 apresentamos os dados sobre acesso e aspectos clínicos. Analisando a forma de acesso ao serviço de saúde, constatou-se que a maioria ingressou pelos fluxos de saúde pública do Sistema Único de Saúde — SUS (77,6%), enquanto os de cor da pele branca referem de maneira significativa que a forma de acesso foi por encaminhamentos de médicos particulares ou de convênio, indicação de pacientes em tratamento no serviço e busca espontânea (31,5%); os autorreferidos pardos/pretos seguiram o fluxo SUS, tendo em maior proporção encaminhamentos de instituições públicas (83,5%).

Entre os participantes do estudo, os estágios clínicos mais prevalentes foram assintomáticos (CDC-A) com 40,6% e AIDS (CDC-C) com 41,4%. As médias (DP) da dosagem de linfócitos CD4 e da carga viral na última consulta foram de 675,5 cel./mm^3^ (492), e carga viral de 45,2 cópias /mL de sangue (12,6), respectivamente. Considerou-se carga viral indetectável <50 cópias de RNA/mL.

Dos participantes, 98,9% estavam em tratamento, tomando em média (DP) 2,9 (2,0) antirretrovirais ao dia. Entre os medicamentos e esquemas de tratamento mais prevalentes encontramos tenofovir/lamivudina/efavirenz (TDF+3TC+EFZ) com 36,9%, tenofovir/lamivudina/atazanavir/ritonavir (TDF+3TC+ATV/R) com 19,1%, darunavir/ritonav (DRV/R) com 21,4%, mas sempre associados com outro antirretroviral, especialmente com TDF3TC (4,28%), sem diferença por raça/cor referida.

A percentagem de internações por todas as causas não associadas ao HIV no último ano foi de 17,4%. Entretanto a prevalência de internações relacionadas ao HIV foi de 57,4%.

Ao avaliar fatores associados às internações relacionadas com o HIV, encontrou-se que os pacientes que tiveram má adesão ao tratamento apresentaram maior prevalência (73,1%) de internações relacionadas com o HIV.

### Informações sobre preconceitos vivenciados

Entre os participantes, 182 (52,0%) referiram ter sofrido algum tipo de preconceito na sua vida, especialmente em função da infecção por HIV — 121 (34,6%). Entretanto, 175 (50%) não referiram ter sofrido preconceito porque *"ninguém sabe da doença",* quando se trata do diagnóstico. Houve diferenças significativas no preconceito pela raça/cor referida e pelo sexo.

### Qualidade de vida e fatores associados

A média geral±DP da HAT-QoL nesta amostra foi de 78,85 (±11,61). Os domínios com maior média foram *confiança no profissional* (M: 94,45±12,62) e *preocupação com a saúde* (M: 92,10±12,47). Os domínios *preocupações com o sigilo* (M: 43,45±29,46) e *preocupações financeiras* (M: 64,62±30,52) obtiveram médias menores (Tabela 4). Para verificar a validade interna da escala HAT-QoL, foi calculada a consistência interna de cada domínio, encontrando-se concordância de boa a excelente.

**Tabela 4:** Estatística descritiva dos nove domínios da qualidade de vida 36-HAT-QoL, de pacientes atendidos no ambulatório de HIV/Aids, Campinas (SP), 2017–2018.

Variáveis ^a^		Mulher (140)	Homem (205)	Total (345)	Valor-p
		f (%)	f (%)	f (%)	
Função geral					
	Mediana (25,75)	90 (76,6–100)	100 (86,6–100)	93,33 (80–00)	0,00^†^
	Média±DP	83,51 (9,11)	88,73 (18,18)	86,57 (18,72)	0,01^‡^
Satisfação vida					
	Mediana (25,75)	80 (60–95)	85 (70–100)	85,00 (65–100)	0,003^†^
	Média±DP	75,24 (20,71)	80,66 (20,39)	78,41 (20,66)	0,016^‡^
Preocupação com a saúde					
	Mediana (25,75)	95 (75–100)	100 (80–100)	95,00 (80–100)	0,044^†^
	Média±DP	82,79 (22,42)	88,95 (16,72)	86,40 (19,49)	0,003^‡^
Preocupação financeira					
	Mediana (25,75)	60 (40–93,3)	80 (40–100)	60,00 (40–100)	0,010^†^
	Média±DP	59,95 (29,47)	67,93 (30,91)	64,62 (30,54)	0,016^‡^
Preocupação com os medicamentos					
	Mediana (25,75)	96 (84–100)	100 (92–100)	96 (88–100)	0,002^†^
	Média±DP	88,75 (15,26)	94,47 (9,38)	92,10 (12,47)	0,001^‡^
Aceitação do HIV					
	Mediana (25,75)	100 (80–100)	100 (100–100)	100 (100–100)	0,003^†^
	Média±DP	84,13 (26,62)	91,70 (20,18)	88,57 (23,33)	----
Preocupação com o sigilo					
	Mediana (25,75)	20 (20–52)	28 (20–72)	24,0 (20–68)	0,022^†^
	Média±DP	38,92 (26,99)	46,65 (30,76)	43,45 (29,46)	0,015^‡^
Confiança profissional					
	Mediana (25,75)	100 (93,3–100)	100 (93,3–100)	100 (98,3–100)	----
	Média±DP	94,94 (12,27)	94,11 (12,86)	94,45 (12,61)	0,546^‡^
Função sexual					
	Mediana (25,75)	100 (40–100)	100 (100–100)	100 (70,0–100)	----
	Média±DP	73,12 (34,01)	89,17 (28,93)	83,17 (28,93)	0,001^‡^
Qualidade de vida geral					
	Mediana (25,75)	76,5 (69,4–83,5)	82,4 (77–87,1)	80,58(72,4–86,5)	0,001^†^
	Média±DP	75,18 (11,81)	81,27 (10,85)	78,85 (11,61)	0,001^‡^

HIV: Vírus da imunodeficiência humana; DP: desvio padrão.

* Distribuição não normal — Shapiro-Wilk (0,001),

p das discretas: Kruskal-Wallis^†^ e t de Student^‡^. ----Não foi possível calcular.

As variáveis associadas a cada domínio e à QV em geral são apresentadas na Tabela 5. No primeiro domínio, que trata de *"Atividades ou Função Gerais"*, observou-se que idade, renda e ter sofrido preconceito estão associados ao domínio. Os pacientes jovens, com renda maior e que não sofreram preconceito, têm índices melhores neste domínio.

**Tabela 5:** Fatores associados com os domínios da qualidade de vida 36-HAT-QoL na análise de regressão de Poisson-Tweedie entre pacientes HIV/Aids, Hospital das Clínicas, Universidade Estadual de Campinas, Campinas (SP), 2018.

Domínios	Variável	Análise bruta		Análise multivariada		
		β	Valor-p	*Adjusted* β	IC95%	Valor-p
Função geral	Idade	-0.002	0,060	-0,002	0,004 a 0,000	0,028
	Sexo (feminino)	-0,053	0,037			
	Renda	0,000	0,003	0,001	-0,006 a −0,005	0,014
	Anos escolaridade	0,008	0,002			
	Internações (sim)	-0,091	0,042			
	Preconceito (sim)	-0,096	0,001	-0,096	-0,144 a −0,047	0001
Satisfação com a vida	Sexo (feminino)	-0,065	0,029	-0,001	-0,003 a −0,046	0,001
	Renda	-0,001	0,003	0,001	0,008 a 0,005	0,052
	Religião (sim)	-0,096	0,019	-0,095	-0,175 a −0,016	0,025
	Moradia*	-0,082	0,001	-0,341	0,534 a −0,144	0,001
	Preconceito (sim)	-0,072	0,013			
	Valor CD4	0,001	0,007	0,001	0,005 a 0,001	0,001
	Internações (sim)	-0,013	0,010	-0,096	-0,191 a −0,001	0,047
Preocupação pela saúde	Sexo	0,063	0,013	0,081	0,027 a 0,137	0,003
	Moradia*	0,050	0,002	-0,111	-0,201 a −0,021	0,016
	Valor CD4	0,001	0,022	0,006	0,001 a 0,005	0,014
	Preconceito (sim)	0,075	0,001	-0,052	-0,106 a 0,001	0,054
	Internações (sim)	0,116	0,007	0,087	0,006 a 0,167	0,034
	Esquema 3/1 (não)	0,066	0,003	-0,050	-0,095 a −0,005	0,029
Preocupação financeira	Sexo (feminino)	0,131	0,012	-0,104	-0,003 a 0,005	0,039
	Cor da pele	0,026	0,001	0,020	0,009 a 0,030	0,001
	Anos escolaridade	0,367	0,001	0,197	0,001 a 0,394	0,049
	Moradia*	-0,324	0,001	-0,263	-0,429 a −0,098	0,002
	Situação laboral^†^	0,224	0,001	0,206	0,111 a 0,301	0,001
	Preconceito (sim)	0,005	0,190	0,005	0,000 a 0,000	0,050
Preocupação com o medicamento	Sexo (feminino)	-0,051	0,001	-0,048	-0,078 a −0,018	0,002
	Preconceito (sim)	0,024	0,052			
	Má adesão (sim)	0,051	0,008	0,051	0,014 a 0,088	0,006
Aceitação do HIV	Idade	0,001	0,248			
	Sexo (feminino)	-0,074	0,015	-0,100	-0,193 a −0,007	0,036
	Anos escolaridade	0,010	0,001			
	Preconceito sexo	0,086	0,044	0,019	0,010 a 0,028	0,001
	Internações	-0,108	0,023	0,155	0,030 a 0,279	0,015
Sigilo	Sexo	-0,209	0,047	-0,198	-0,120 a −0,010	0,042
	Estado civil	-0,216	0,047			
	Preconceito OS^‡^	-0,239	0,036	-0,313	-0,541 a −0,084	0,007
	Valor carga viral	-0,001	0,001	-0,001	-0,003 a −0,001	0,023
	Tempo diagnóstico	-0,013	0,117	-0,033	-0,060 a −0,006	0,017
	Tempo tratamento	0,003	0,051	0,047	0,020 a 0,074	0,001
Confiança no profissional	Anos escolaridade	-0,004	0,024			
	Estágio da doença	-0,062	0,001			
	Preconceito	-0,037	0,024	-0,071	-0,067 a −0,036	0,029
	Tempo tratamento	0,003	0,013	-0,035	-0,036 a −0,004	0,001
	Valor CD4	0,005	0,145	0,005	0,006 a 0,005	0,027
Atividade sexual	Idade	-0,004	0,050			
	Sexo	-0,211	0,001			
	Escolaridade	0,019	0,001	0,214	0,120 a 0,309	0,001
	Renda	0,328	0,032	0,016	0,005 a 0,027	0,005
	Orientação Sexual^§^	0,134	0,020			

HIV: Vírus da imunodeficiência humana.

* Moradia cedida ou ocupação;

^†^Desempregados; ^‡^Preconceito por orientação sexual (OS); ^§^Homossexual.

O segundo domínio, denominado *"Satisfação com a Vida",* está associado com sexo, renda, religião, tipo de moradia, valor do CD4 e internações no último ano. Pacientes do sexo masculino, com melhor renda mensal, que praticam alguma religião e possuem moradia própria, não tiveram internações no último ano e exibiram valores de CD4 acima de 200 cel./mm^3^ apresentaram melhor satisfação com a vida.

O terceiro domínio, *"Preocupação com a Saúde"*, foi associado às variáveis sexo, tipo de moradia, preconceito, internações e esquema de tratamento TDF+3TC+EFZ (3/1). Os pacientes de sexo feminino, que vivem em moradias cedidas ou em ocupação, que sofreram algum preconceito, têm CD4 menor de 200, foram internados no último ano e não estão em tratamento com TDF+3TC+EFZ apresentam maior preocupação pela sua saúde.

No quarto domínio, que trata da *"Preocupação Financeira"*, encontrou-se média (DP), marcando moderada qualidade de vida. Este domínio foi associado ao sexo, anos de escolaridade, tipo de moradia, situação laboral, preconceito e valor do CD4. As mulheres, pacientes com baixa escolaridade, desempregados, sem moradia própria, que já sofreram preconceito especialmente por raça e que têm contagem de CD4 abaixo de 200 apresentam maior preocupação neste aspecto.

No quinto domínio se avaliam *"Questões relacionadas à Medicação"*. Os quatro itens foram respondidos por 346 pacientes que utilizavam os antirretrovirais; os quatro que não recebiam medicação eram pacientes nomeados como Controladores de Elite. Apenas as variáveis sexo e adesão ao tratamento se encontraram associadas: mulheres e pacientes com má adesão têm maior preocupação com os efeitos dos medicamentos.

A *"Conscientização ou Aceitação do HIV"* foi avaliada no sexto domínio, tendo como fatores associados sexo, anos de escolaridade e preconceito pelo sexo. As mulheres, os pacientes com baixos níveis de escolaridade e os que sofreram preconceito apresentam menores níveis de aceitação da doença.

A média (DP) do sétimo domínio, que trata da revelação do sigilo sobre a infecção pelo HIV, foi de 43,45 (29,46) pontos, sendo considerada baixa. Encontraram-se associados ao sexo o preconceito pela orientação sexual, o tempo de diagnóstico e o tempo de tratamento. Pacientes de sexo feminino, os homossexuais, os que tinham menor tempo de diagnóstico e tratamento apresentavam maior medo de que o diagnóstico do HIV fosse conhecido pelos outros.

O oitavo domínio investiga *"Confiança Profissional"*, com média mais alta. A descrição deste domínio, na escala original, mostra que ele pode ser utilizado para avaliar qualquer dos profissionais da equipe de saúde. Neste estudo, optou-se por avaliar o relacionamento com o médico por ser a consulta médica a mais regular e sistemática no seguimento ambulatorial. O estágio da doença (CDC-B e C), o preconceito e o tempo de tratamento estão associados a este domínio.

O nono domínio, intitulado *"Atividade Sexual*", obteve índice médio (DP) de 83,1 pontos (20,0–100,0). Pelos resultados da análise multivariada, verificou-se associação das variáveis sexo feminino e baixa escolaridade com o menor escore.

## DISCUSSÃO

Verificamos que os domínios mais afetados da QV na população avaliada foram a SV, as preocupações PF e PSi (sigilo), como descrito em outros estudos^12,13^. Esses domínios foram associados, respectivamente, às variáveis: sociodemográficas (sexo feminino, renda, moradia) e clínicas (valor de CD4 e internações); sociodemográficas (incluindo raça e situação laboral) e ter sofrido preconceito; e preocupações com sigilo, sexo, preconceito e variáveis clínicas (valor de CD4 e tempos de diagnóstico e tratamento).

Embora o objetivo do estudo fosse avaliar as diferenças da QV de pacientes HIV/AIDS por raça/cor da pele autorreferida, ao desenvolver a pesquisa pudemos verificar que os participantes de raça negra apresentam menor escolaridade e renda, estão em maior proporção desempregados e moram em casa alugada ou cedida quando comparados com os de cor da pele branca. Todavia, as diferenças na QV de vida estão marcadas pelo sexo, pela renda e pela escolaridade, o que acompanha o descrito por vários autores, que afirmam que no Brasil a epidemia se disseminou entre os moradores de comunidades mais pobres, especialmente entre as mulheres^14^, entre os de menor educação formal^10^ e entre aqueles inseridos em ocupações mal remuneradas ou excluídos do mercado de trabalho formal^10,15^. Ou seja, progressivamente, a epidemia passou a refletir os padrões de desigualdade predominantes no país^16^.

Os achados confirmam que as desigualdades de gênero constituem um dos principais impulsionadores estruturais subjacentes da equidade em saúde, como descrito pela Comissão sobre Equidade e Desigualdades em Saúde nas Américas^17^. Na pesquisa, tanto renda e escolaridade como os níveis de vários domínios da QV têm menores valores no sexo feminino quando comprados com os do sexo masculino. Com relação aos índices da QV, nossos dados assemelham-se a estudo realizado entre mulheres portadoras de HIV, que aponta prejuízo nos domínios PF, PS, PSi e AS^13^; porém não foi encontrada associação com SV, AHIV e PM, como no presente estudo. Entretanto, estudo desenvolvido em Hospital municipal em Rio Grande do Norte, ao avaliar esta variável (sexo), encontrou diferenças nos domínios PF, PM e AS^12^ entre as mulheres.

Contudo, ainda os pacientes de raça negra tiveram níveis baixos de QV só no domínio PF; e é fundamental reforçar as dificuldades que esse grupo apresenta no acesso ao diagnóstico e tratamento, pois esses indivíduos acessam principalmente pelos fluxos da rede de saúde pública, mas especialmente pelo pronto-atendimento/emergência, chegando em piores condições de saúde que os pacientes de cor da pele branca. O retardo no acesso ao serviço especializado e no início do tratamento possibilita complicações clínicas e maior risco de mortalidade, como descrito em outros estudos^18,19^. Adicionalmente, observa-se que a proporção de pacientes com condição clínica CDC C (AIDS) entre este grupo (32,9%), as internações no último ano (11,1%) e a causa delas (HIV) (38,3%) é maior entre os de cor da pele parda/preta quando comparamos com os de cor da pele branca. Fica marcada a determinação social no percurso recorrido pelos pacientes de raça negra para conseguir tratamento e QV adequados, especialmente sendo portadores do HIV.

Como é afirmado por Albuquerque et al., a desigualdade racial tem sido fator de influência e/ou determinação do lugar do indivíduo na sociedade, definindo seu acesso, maior ou menor, à riqueza, escolaridade, moradia, bens públicos, serviço de saúde, informação, entre outros^20^. Neste estudo, pode-se visualizar essas desigualdades particularmente na população negra e feminina.

Segundo Galvão et al., a literatura destaca como o racismo opera não apenas de forma explícita, mas também em mecanismos subliminares que geram efeitos cumulativos e duradouros^21^. O racismo estrutural, cristalizado e internalizado na cultura de um povo, exclui uma parcela da população do acesso à educação, emprego, mobilidade social ascendente, informação e, por conseguinte, acesso à saúde. Já o racismo institucional é resultado de políticas, práticas e procedimentos de instituições que causam efeito negativo no acesso das minorias raciais, como também na qualidade de bens, serviços e oportunidades^22^. Embora a ciência tenha avançado no conhecimento sobre iniquidades em saúde, as experiências de racismo ainda são subestimadas.

Corroborando nossos achados, a QV das PVHIV não está apenas relacionada com a possibilidade de uma vida mais longa, pois o enfrentamento da doença torna-se cada vez mais problemático, principalmente quando se trata da falta de recursos sociais e financeiros^16^. No domínio PF, investigou-se a preocupação em ter que viver de renda fixa, em ter que acertar as contas, ou mesmo simplesmente ter ou não a oportunidade de ter lazer ou alguma satisfação com sua renda mensal^23^.

Indivíduos em situação de pobreza vivenciam sérias dificuldades de acesso ao tratamento, o que pode trazer prejuízos para viver com HIV^24^. Nosso estudo averiguou que os participantes da amostra estão em situação de limitação financeira e em maior nível os de raça negra e as mulheres, reforçando as diferenças socioeconômicas.

O domínio PM investiga se o fato de os pacientes terem que tomar os medicamentos os faz sentirem-se mais doentes ou mais saudáveis quando percebem que estão combatendo o vírus. Também investiga se as drogas interferem na vida normal (efeitos colaterais)^25^. A adesão inadequada ao tratamento com antirretrovirais está em alguns casos associada com o descrito em estudo realizado na Tailândia, com a possibilidade de revelação da condição sorológica pelo uso dos medicamentos^26^. No presente estudo, observamos associação entre os domínios PM e PSi.

O domínio que trata da divulgação do sigilo sobre a infecção pelo HIV é avaliado como a preocupação e/ou a limitação com relação à revelação do diagnóstico da infecção para familiares e colegas de trabalho, bem como a preocupação de que as pessoas descubram seu diagnóstico^27^. Na amostra avaliada, 50% dos pacientes relataram que "ninguém sabe da doença", o que justifica a baixa pontuação apresentada, mas mostra-se como uma estratégia para se proteger dos preconceitos que ainda existem em nossa sociedade. Logie et al.^28^ registram que o estigma relacionado ao HIV e a discriminação racial e de gênero estão correlacionados e comprometem a saúde física, mental e a QV deste grupo, como descrito no presente estudo.

Assim como em outros estudos, observou-se que a confiança no profissional está mais centrada na figura do médico. Pesquisas sugerem que a figura do profissional médico pode levar o paciente a manter-se bem fisicamente, a desenvolver as diversas atividades sugeridas e a ter melhor QV^13^. Entretanto, Guibu et al.^29^ mostram que o acesso à equipe multiprofissional está relacionado com a maior sobrevida dos pacientes com Aids nas Regiões Sul e Sudeste do Brasil.

Estudos mostram que a interferência na QV dos pacientes com HIV é multifatorial. O sigilo sobre a doença, condições socioeconômicas como trabalho e renda, estresse emocional, intensificado pela discriminação e pobreza, impactam negativamente diversas esferas da vida dos indivíduos, o que coincide com os achados neste estudo^24,25^.

Cabe enfatizar que, entre as limitações da pesquisa, encontramos seu delineamento transversal, que impede a identificação de causalidade; a amostra por captação sistemática semanalmente e o uso de variáveis autorreferidas, que podem comprometer a representatividade apresentando vieses de seleção e de informação. Adicionalmente, a ausência de controle para variáveis contextuais e a coleta restrita a um único ambulatório limitam o alcance dos achados e sua generalização.

As desigualdades observadas em pessoas que vivem com HIV/Aids neste estudo são semelhantes às encontradas em várias partes do mundo. Podemos concluir que, mesmo diante de políticas públicas muito bem definidas de atenção integral à saúde, que reforçam o acesso universal à terapia antirretroviral^1,30^, ainda existe diferença no acesso ao tratamento e na QV por sexo e cor da pele neste grupo.

## Footnotes

1 Foi escolhido o Hospital Dia porque é ligado ao Hospital das Clínicas da Unicamp e atende pacientes que vivem com HIV/Aids e doenças infectocontagiosas, sendo referência na região.
